# Correction: Nuclear analytical techniques used to study the trace element content of *Centaurium erythraea* Rafn, a medicinal plant species from sites with different pollution loads in Lower Silesia (SW Poland)

**DOI:** 10.1371/journal.pone.0314406

**Published:** 2024-11-20

**Authors:** Anna Brudzińska-Kosior, Grzegorz Kosior, Monika Sporek, Zbigniew Ziembik, Inga Zinicovscaia, Marina Frontasyeva, Agnieszka Dołhańczuk-Śródka

The affiliation for the third author is incorrect. The correct affiliation is not indicated. Monika Sporek is not affiliated with #2 but with: Institute of Biology, University of Opole, Opole, Poland.
