# Supplementary material for: Nuclear analytical techniques used to study the trace element content of Centaurium erythraea Rafn, a medicinal plant species from sites with different pollution loads in Lower Silesia (SW Poland)
Source: PLoS One. 2023 May 18;18(5):e0285306. doi: 10.1371/journal.pone.0285306 (PMC10194893; doi:10.1371/journal.pone.0285306)
Supplement: Attachment — Submitted filename: Nuclear analytical techniques C. erythraea Reviewed by Milana Trifunovic-Momcilov.doc [file pone.0285306.s001.doc]

Nuclear analytical techniques used to study trace elements content of Centaurium erythraea Rafn. medicinal plant from sites with different pollution load in the Lower Silesia (SW Poland)

# Anna Brudzińska-Kosiora, Grzegorz Kosiora, Monika Sporeka, Zbigniew Ziembika, Inga Zinicovscaiabc, Marina Frontasyevab, Agnieszka Dołhańczuk-Śródkaa,

^a^Institute of Environmental Engineering and Biotechnology, University of Opole, ul. kard. B. Kominka 6, 45-032 Opole, tel: +48/781797137

^b^Department of Neutron Activation Analysis and Applied Research, Division of Nuclear Physics, Frank Laboratory of Neutron Physics, Joint Institute for Nuclear Research, Moscow Region, Russian Federation

^c^Horia Hulubei National Institute for R&D in Physics and Nuclear Engineering, Bucharest - Magurele, Romania

*Corresponding author during period of submission:

# **Grzegorz Kosior grzegorz.kosior@uni.opole.pl**

**Abstract**

*Centaurium erythraea* (Gentianaceae) is a plant with therapeutic potential officially listed in pharmacopoeias of many European, Asian and American countries. This plant has many uses in natural medicine since ancient times. This plant is collected mostly from wild populations. The aim of this study is to investigate the trace elements composition in *C. erythraea* by the method of instrumental neutron activation analysis (INAA). The results of performed investigations show that INAA has proved to be an efficient analytical technique for determination of trace elements in medicinal plants. The studied plant - *C. erythraea* contain elements of importance for human diet and metabolism and that are needed for growth and developments, prevention and healing of diseases. A comparison with the “reference plant” shows that concentrations of most elements in *C. erythraea* collected from all types of sites exceed those regarded as reference. Compared to the values of elements in *C. erythraea* from rural areas (LP), the concentrations of most of the investigated elements in *C. erythraea* collected from lignite basin, urban areas and vicinity of A4 highway (MP) were significantly higher. The results obtained that INAA can be used for control and monitoring in production of pharmaceutical on the base nature medical plants.

**Introduction**

The use of medical herbs to relieve and cure human diseases was in wide use in former times, but their applications decreased with introduction of modern medicine. Recently use of medicinal plants is increasing again around the world due to their features and low side effects (Juranović Cindrić et al. 2013; Shikov et al., 2014; Ziener et al., 2015). A significant interest in herbal remedies and preparations causes increasing demand for medicinal plant raw materials (Shikov et al., 2014). Despite the serious degradation of the environment resulting from a dynamic development of industry in Europe and all over the world, the collection of plant material from natural habitats still remains the main source of plants commonly used in natural medicine (Piatczak et al. 2005, Suchacz and Wesołowski 2006). Knowledge of the elemental content of medicinal plants is very important because some of the elements are closely related to essential set of elements in human body (Baljinnyama et al., 2014). On the other hand, the interest in chemical composition of medicinal herb products is growing because of ongoing developments in nutrition and in biochemical surveying and mineral prospecting (Rodushkin et al., 1999). Therapeutic properties of numerous herbal plants are well documented however very little information is available regarding their trace element contents. It is therefore imperative to monitor these herbs for the presence of necessary and toxicant elements levels prior to their direct use in phytotherapy by consumers or in formulations in the pharmaceutical industry to determine their efficacy and safety (Desideri et al., 2010).

*Centaurium erythraea* (Gentianaceae) is a plant with therapeutic potential (Piatczak et al. 2005; Shikov et al., 2014). This species is a widespread plant in Europe and parts of western Asia and northern Africa. *C. erythraea* occurs mostly in open habitats with bare areas, such as dry calcareous grasslands and early-successional stages of forest colonisation, i.e. after clear-cutting, in forest edges and clearings, and in open woods (Van Rossum, 2009). This plant has many uses in natural medicine since ancient times (Stefkov et al., 2014). Extract of the aerial parts of the plant is used as stomachic and bitter tonic for digestive complications and to treat febrile conditions, diabetes, heartburn (Leclerc 1976, Stefkov et al., 2014). 'Centaurii herba' is also used in the natural traditional medicine to cleanse blood and kidneys, relieve indigestion, heal wounds and sores (Kumarasamy et al., 2003, Hamza et al., 2015). This plant is collected mostly from wild populations (Piatczak et al. 2005; Subotić et al. 2009; Shikov et al., 2014). In Poland there are collected about 10 tons of raw material of this plant for a year (Brudzińska-Kosior et al. 2012). This is a large amount because the plant is relatively small, and the number of sites of its occurrence is very limited. Frequently this plant is collected from all available sites of occurrence. Natural habitats of this species may be influenced by trace elements pollution from local or long-range deposition (Brudzińska-Kosior et al. 2012). Therefore, there is a need to study the biogeochemistry of *C.erythraea* to determine doses in case of ingestion as a remedy and to determine if this species is an accumulator of trace elements (Ražić et al., 2003).

The aim of this study is to investigate the trace elements composition in *C. erythraea* by the method of instrumental neutron activation analysis (INAA). Total concentrations of the elements: Al, As, Ba, Br, Ce, Cl, Co, Cr, Cs, Fe, Hf, La, Mn, Mo, Ni, Rb, Sb, Sc, Se, Sm, Sr, Tb, Th, U, V, W and Zn were determined in raw material from regions with different pollution levels: lignite mining industry with lignite-fired power plant, urban and rural sites. Another significant aspect of the study is to classify the results and to examine whether or not there is a relationship between the trace elements by data mining analysis. To achieve this scope, the covariabilities to describe random or non-linear relationship between concentrations, principal component analysis and cluster analysis method were used. Results from this study allow to specify dosage for medicines prepared from *C. erythraea* and the degree of a risk for elements considered as harmful to consumers.

The tested hypothesis: (1) whether *C. erythraea* from sites with different anthropogenic loading is contaminated by elevated concentrations of selected trace elements causing risk for medicinal consumption; (2) whether statistical techniques separating the concentration of elements in this species will allow to distinguish a pattern and a relationship between the trace elements.

**Materials and methods**

## Sampling design

In the Lower Silesia 10 sampling sites of *C. erythraea* were selected: sites (1-3, 9) in the polluted area of Bogatynia lignite basin; sites (6-8) were located near the urban areas of Wrocław city influenced mostly by urban pollutants; sites 4 and 5 were located in rural areas near Bolesławiec; site 10 was located in vicinity of A4 highway this area is affected by transport pollutants (Fig. 1). At each site, five samples of leaves of *C. erythraea* were collected randomly within a 25 m x 25 m square. Each sample consisted of a mixture of three subsamples. The total number of plant samples was n = 10 x 5 = 50. The collected plants had not been exposed directly to canopy through fall. Leaves were transported to the laboratory wrapped in aluminum foil.

**Samples collection and preparation**

In the laboratory the sample of *C. erythraea* was cleaned from extraneous plant materials and dried at room temperature and then oven dried at 40° C for 48 hours until constant weight. The samples were not washed. The dried samples were ground into fine powder in an agate mortar. Two sub-samples of each plant were pressed into thick pellets using a press-form of 15 mm diameter in without binder. Pellets of plants of ~ 0.3 g were heat-seated in polyethylene foil bags and packed in aluminum cups for short and long irradiation, respectively (Baljinnyama et al., 2014).

**Analysis**

The concentration of elements in the herbal plant samples was determined by a multi-element instrumental neutron activation analysis (INAA) at the IBR-2 reactor, FLNP JINR, Dubna. The detailed procedure of samples irradiation can be found in (Baljinnyam et al.,2014, Zinicovscaia et al. 2019, 2020).

Two types of irradiations were done. The concentrations of elements based on short-lived radionuclides: Al, Cl, Mn, and V were determined by irradiation for 3 min and spectra measurement for 15 min. To determine elements with long lived isotopes: As, Br, Fe, La, Mo, Sm, U, W, Ba, Ce, Co, Cr, Cs, Hf, Ni, Rb, Sb, Sc, Se, Sr, Tb, Th, and Zn the cadmium-screened channel 1 was used. Samples were irradiated for 4 days at a neutron flux of 1.8×10 ^11^ cm^−2^ s^−1^·min, re-packed and measured twice, first in 4 days for 30 min and then in 20 days for 1.5 hours. Gamma spectra of induced activity were obtained using three spectrometers based on HPGe detectors with an efficiency of 40-55% and resolution of 1.8-2.0 keV for total-absorption peak 1332 keV of the isotope ^60^Co.

The analysis of the spectra was performed using the Genie2000 software from Canberra, with the verification of the peak fit in an interactive mode. The calculation of concentration was carried out using software “Concentration” developed in FLNP (Pavlov et al., 2016).

## Quality Control

The quality control of the analytical measurements was carried out using certified reference materials: NIST SRM 1573 - Tomato leaves, NIST SRM 1575a - Pine needles, and NIST SRM 1547 – Peach leaves, NIST SRM 1633b - Coal fly ash, IRMM BCR-667- Estuarine sediment and NIST SRM 2710 - Montana Soil were irradiated under the same experimental conditions as the samples. Good agreement between experimental results and certified values was obtained (Table 1).

## Statistical analysis

The compositional data are not independent on each other, if content of one of components increases the others have to decrease. It is the reason for which results of standard statistical analysis of the relationships between raw components or parts are spoiled by spurious effects (Pawlowsky-Glahn and Buccianti 2011; Pawlowsky-Glahn and Egozcue 2006). The particular properties of compositional data preclude the application of standard statistical techniques on such data in raw form. For example, variances of constrained, compositional data components are not independent. Variance in a component concentration may not be strictly related to variance in the component abundance, because this variability is affected also by variabilities in abundances of other components. As the result of the closure operation, variance of a component and its covariances with the other component are bound with the relationship:

Application of classical data analysis methods for statistical analysis of compositional data may lead to false or delusive conclusions. This is why in data interpretation statistical methods designed for compositional data elaboration were used. Particularly, utilization of classical correlation estimator in statistical analysis of compositional data can lead to delusive conclusions (Filzmoser and Hron 2008). Alternative method, appropriate for covariability estimation in compositional data, is based on variations matrix **T**. Elements *t* of this matrix are variances of concentration pairs logratios:

Variance of logratio is small when changes in both concentration follow the same trend, ie. both values simultaneously increase or decrease. The biggest logratio variance is observed when concentrations follow opposite trends. Increase in one variable values accompanied with decrease in values of the second one produces highly differentiated logratios. As the result, the biggest *t* value is observed. To distinguish between these two types of covariability, terms “positive” (increase in both concentrations) and “negative” (increase in one concentrations and decrease in the second) is used.

The *t* values between these characteristic for negative or positive covariabilities describe random or non-linear relationship between concentrations. The intermediate values of randomly arranged data can be used in assessment of actual *t* classification.

The distribution function of *t* can be estimated on the base of the actual data. Data permutation in the first variable, keeping unchanged sequence in the second one, produces a number of *t* values. Most of them correspond to random arrangement in variables pairs. A fraction of the lowest correspond to concentrations positively related with each other. A fraction of the highest *t* values correspond to negative covariability between concentrations (Ziembik and Dołhańczuk-Śródka 2015). In this work the *t* distribution quantile *q*_0.01_ was the upper limit for the positive covariability, and *q*_0.99_ was the lower limit for the negative one. Though, as it was mentioned earlier, Pearsons correlation coefficient *r* generally is not appropriate for covariability assessment among concentrations, its value was calculated to compare conclusions with the ones related to the *t* estimates.

In computations the R language (R Development Core Team 2015) and functions from libraries “cluster” (Kaufman and Rousseeuw 2005; Maechler et al. 2014) and “compositions” (van den Boogaart et al. 2014) were used. Concentrations of elements in *C. erythraea* from more polluted (MP - lignite basin, urban areas and vicinity of A4 highway) and less polluted (LP - rural areas) were examined with the t-test applied to the log-transformed data.

## Results and discussion

The concentrations of trace elements determined in *C. erythraea* are given in (table 2). The statistical parameters of the data were calculated. In table 2 the following parameters are shown: min– minimal value, q_1_– lower quartile, median, arithmetic mean, q_3_– upper quartile, max – maximal value, SD – standard deviation, and MADN – normalized median absolute deviation about the median (Maronna et al., 2006). The MADN parameter can be regarded for robust alternative of SD. If no outliers are in vector of normally distributed data then MADN and SD values are similar to each other. Comparison of median and mean values of concentration indicates skewness in data distribution. For Cl, Fe, Rb and Zn mean to median ratio exceeded 1.5. These results suppose outlying observations in the data. Compared to the average concentrations given by Markert (1992) for terrestrial plants (limits in mg kg^-1^ in parentheses) the upper concentrations of the following elements were within the ranges: Ba, Cs, Mn, Ni, Rb, Sb, Sr and Tb in *C. erythraea* collected from all sites (table 2). The highest concentrations of elements in plant were determined in *C. erythraea* collected from sites affected by lignite industry and from urban areas except for Br, Cl, and Mo (the highest concentration in plants from rural areas). To assess the linear covariability between pairs of elements concentration, quantiles of the t parameter distribution were calculated. In table 3 the results of computations are shown. Symbols describe the covariability character, i.e.: ‘r’ is for random or no linear relationships between concentrations, ‘+’ is for positive covariability and ‘-‘ is for negative covariability. For comparison, the commonly used in data interpretation Pearson's correlation coefficients r were calculated. The r parameters were tested for 0 value in population, on 0.02 *p*-level. For 2D normally distributed data composed of variable pairs representing 30 cases the critical, absolute |*r*_c_| value is 0.423. In table 3 the positive correlations bigger than r care in bold and the negative correlations smaller than r care underlined. Positive covariability was obtained for the pairs of elements originated from anthropogenic sources and from substrate. For the elements that are regarded as microelements in the human diet (Co, Cr, Fe, Mn, Se, V and Zn) were found positive covariability for multiple elements (table 3). Negative covariabilities were obtained for only 4 pairs of elements (including 2 with Cl).

In recent years, there is increasing interest in determination of rare earth elements (REEs) content in medicinal plants, as are they able to affect the growth and/or development of plants directly or indirectly (Zhang et al., 2013). INAA possess the unbeatable position in the determination of REEs. In this study, the concentrations of following REEs were determined: Ce, La, Sc, Sm and Tb. The average concentration of these elements for all sites is higher than the reference plants values (Markert 1992). However, it must be noted that average concentrations of REEs are mainly influenced by plants collection place to a greater extent by sites located near the lignite industry and to a lesser extent, by sites located in the urban areas (table 3). Concentrations of REEs can exert positive or negative physiological effects on plants depending on the dosage and other conditions. However, if the concentration of REEs exceeds the optimum level, they can inhibit the plant growth and even cause mortality (Zhang et al., 2013). It is believed that REEs can regulate absorption of other mineral elements in plants. In some cases, the lowest *t* value in the complete data (positive covariabilities, increase- increase) was found for pairs of the rare earth elements such as La – Sm, La – Tb, Sm – Tb (table 3). Compared to the values of elements in *C. erythraea* from rural areas (LP), the concentrations of most of the investigated elements in *C. erythraea* collected from lignite basin, urban areas and vicinity of A4 highway (MP) were significantly higher (test t, P < 0.05) (Table 4).

In this study content of trace elements (Co, Cr, Fe, Mn, Mo, Ni, Se, V and Zn) regarded as microelements in the human diet was determined*.* The total level of these elements in the plant permit us to conclude that, *C. erythraea* can be considered as an important source of these elements for humans (especially Co, Fe, Se, V and Zn), even after preparing infusions using raw plant material. These elements play an important role in mineral nutrition of humans. For instance, Fe is useful in prevention of anemia and other related diseases. It is needed for healthy immune system and energy production also (Rajan et al., 2014). Co is also essential for human health is a key constituent of cobalamin, also known as vitamin B12 and deficiency of it results in the less production of red blood cells in the body necessary for the rapid transport of oxygen to injured cells (Cracan and Banerjee 2013) Although the biological role of V is limited and available reported literature is mostly on the beneficial role in the management of diabetes mellitus, the function of V will be probably part of the protection system against injury of tissues (Rajan et al., 2014). Zinc is useful for protein synthesis, normal body development and recovery from illness (Basu et al., 2014). Particular attention should be paid to selenium, because Se is an important element in the treatment of human tumors. Depending on concentration and chemical form, Se may prevent or treat tumor disease. The inhibitory effects are tumor specific and selenium induces apoptosis in malignant cells at concentrations that do not affect the viability of normal cells ([Björnstedt](http://www.ncbi.nlm.nih.gov/pubmed/?term=Bj%26%23x000f6%3Brnstedt M%5Bauth%5D) and Fernandes 2010). Se levels in *C. erythraea* from all studied sites (0.3 mg kg^-1^) was significantly higher compared to the average concentrations given by Markert (1992) for terrestrial plats, therefore *C. erythraea* can be considered as relatively rich in Se. However, the average concentrations in plants collected from lignite industry sites was 0.5 mg kg^-1^. The daily intake of medicinal plant can cause overdose of Se (recommended daily dietary allowance is 0.02-0.2 (Ivey et al., 1986). For human zinc, iron, copper, chromium, molybdenum, selenium and cobalt are besides others considered as essential and become harmful only at high concentrations (Gupta 2014). Amidst the tendency of people to consume health promotion foods based on their distrust of medicines, preparations of trace elements are available commercially, and inappropriate use of these preparations can cause excess states of these trace elements (Wada, 2004). Persons more susceptible to harmful effects of uptaken low doses of heavy metals by diet, like children, pregnant or elderly people might show adverse reactions (Zeiner et al. 2015). It should be emphasized that the sites contaminated achieved a higher concentration of these elements, but they are not dangerous levels except lignite sites. Unfortunately, the maximum allowable levels of elements in medicinal plant materials and finished herbal products are not regulated. The World Health Organization WHO mentions maximum permissible levels in raw plant materials only for As, Cd and Pb, namely 1.0, 0.3 and 10 mg kg^-1^, respectively (WHO, 1991). In our study we investigated only As, concentration of this element in plants collected from lignite industry site (1.7 mg kg^-1^) exceeded permissible level.

Another reason for undertaking the precise and accurate elemental analysis medicinal plant formulations is the need to assess and monitor the safety and quality of these products (Pohl et al., 2016), primarily in reference to the content of elements that may cause negative effects on the human body, i.e. Al, As, Ba, Cd, Ni, Pb, Sb and Sn. The content of the latter undesirable elements, potentially exhibiting toxic effects, has to be verified. This can lead to avoiding over-consumption and intoxication (Pohl et al., 2016). In this study, the concentrations for these elements were higher than the average for plants except Ba and Sb (Markert, 1992).

### Conclusions

The results of performed investigations show that INAA has proved to be an efficient analytical technique for determination of trace elements in medicinal plants. The studied plant - *C. erythraea* contain elements of importance for human diet and metabolism and that are needed for growth and developments, prevention and healing of diseases. The level of trace elements in plants is affected by the geochemical characteristics of the soil and the ability of plants to selectively accumulate some of the elements. A comparison with the “reference plant” shows that concentrations of most elements in *C. erythraea* collected from all types of sites exceed those regarded as reference. Compared to the values of elements in *C. erythraea* from rural areas , the concentrations of most of the investigated elements in *C. erythraea* collected from lignite basin, urban areas and vicinity of A4 highway were significantly higher. The results obtained that INAA can be used for control and monitoring in production of pharmaceutical on the base nature medical plants.

**References:**

Baljinnyama N., Tsevegsuren N., Jugderc B., Frontasyeva M. V., Pavlov S. S. 2014 Investigation of Elemental Content of Some Medicinal Plants from Mongolia. International Journal of Medicinal Plants. Photon 106 481-492

Subhashree B., Moumita D., Anurupa S. , Utpal R. C., Gouriprosad D. Analysis of complete nutritional profile and identification of bioactive components present in Alocasia indica tuber cultivated in Howrah District of West Bengal, India Asian Pac J Trop Med 2014; 7(Suppl 1): S527-S533

# **Björnstedt Mikael** and [**Aristi P. Fernandes**](http://www.ncbi.nlm.nih.gov/pubmed/?term=Fernandes AP%5Bauth%5D) Selenium in the prevention of human cancers. **EPMA J. 2010 Sep; 1(3): 389–395.**

Brudzińska-Kosior A., Kosior G., Samecka-Cymerman A., Kolon K., Mróz L., Kempers A. J. 2012 Metal contents in Centaurium erythraea and its biometry at various levels of environmental pollution Ecotoxicology and Environmental Safety 80 349–354

Chaturvedi UC, Shrivastava R, Upreti RK. Viral infections and trace elements: a complex interaction. Curr Sci 2004; 87: 1536-1554.

Cracan V, Banerjee R. Cobalt and corrinoid transport and biochemistry. In: Banci L, editor. Metallomics and the cell. Germany: Springer; 2013, p. 12.

Desideri D., Assunta Meli M., Roselli C. 2010 Natural and artificial radioactivity determination of some medicinal plants Journal of Environmental Radioactivity 101 751-756.

Frontasyeva M. V., Pavlov S. S., 2000. Analytical Investigations at the IBR-2 Reactor in Dubna, JINR Preprint, E14-2000-177, Dubna.

S.C. Gupta, Sources and deficiency diseases of mineral nutrients in human health and nutrition: a review, Pedosphere 24 (2014) 13–38.

N. Hamza, B. Berke, C. Cheze, S. Marais, S. Lorrain, A. Abdouelfath, R. Lassalle, D. Carles, H. Gin, N. Moore 2015 Effect of Centaurium erythraeaRafn ,Artemisia herba-alba Asso and Trigonella foenum-graecum L. on liver fat accumulation in C57BL/6J mice with high-fat diet-induced type 2 diabetes. Journal of Ethnopharmacology 171 (2015) 4–11

Ivey M., Elmen G., 1986. Nutritional Supplements, Mineral and Vitamin Production in: Handbook of Nonprescription Drugs, 8th ed., American Pharmaceutical Association, The National Professional Society of Pharmacists, 2215 Constitution Avenue N.W. Washington, DC 20037, USA, 215.

A. Jacob, in: N.W. Tietz (Ed.), Textbook of Clinical Chemistry, Saunders, Philadelphia, PA, 1994, pp. 965-980. [21] S.S.

Juranović Cindrić I., Zeiner M., Glamuzina E., Stingeder G. 2013 Elemental characterisation of the medical herbsSalvia officinalisL. and Teucrium montanum L. grown in Croatia. Microchemical Journal 107 185–189.

N.F. Kolachi, T.G. Kazi, H.I. Afridi, S. Khan, S.K. Wadhwa, A.Q. Shah, F. Shah, J.A. Baig, Sirajuddin 2010 Determination of selenium content in aqueous extract of medicinal plants used as herbal supplement for cancer patients Food and Chemical Toxicology 48 (2010) 3327–3332

Y. Kumarasamy, L. Nahar, S.D. Sarker, Bioactivity of gentiopicroside from the aerial parts of Centaurium erythraea, Fitoterapia 74 (2003) 151–154.

H. Leclerc, Precis de Phytotherapie, Masson, Paris/New York/Barcelone/ Milan, 1976.

Markert, B. (1992). Chapter 15: Multi-element analysis in plant materials – analytical tools and biological questions. In D. C. Adriano (Ed.), Biogeochemistry of trace metals (pp. 401–428). Boca Raton: Lewis Publishers.

Ostrovnaya T. M., Nefedyeva L. S., Nazarov V. M., Borzakov S.B., Strelkova L. P., 1993. Software for INAA on the Basis of Relative and Absolute Methods Using Nuclear Data Base, Activation Analysis in Environment Protection, D-14-93-325, 319-326 Dubna.

Ozcan, M. (2004). Mineral contents of some plants used as condiments in Turkey. Food Chemistry, 84, 437–440.

Piatczak E., Wielanek M., Wysokinska H. 2005. Liquid culture system for shoot multiplication and secoiridoid production in micropropagated plants of Centaurium erythraea Rafn Plant Science 168 431–437

Jay Prakash Rajan1, Kshetrimayum Birla Singh2*, Sanjiv Kumar3, Raj Kumar Mishra Trace elements content in the selected medicinal plants traditionally used for curing skin diseases by the natives of Mizoram, India. Asian Pac J Trop Med 2014; 7(Suppl 1): S410-S414

Trace elements analysis of Echinacea purpurea herbal medicinal Slavica Ražić, Antonije Onjia, Branislav Potkonjak Journal of Pharmaceutical and Biomedical Analysis 33 (2003) 845/850

Rodushkin I., Ruth T., Huhtasaari A., 1999. Comparison of two digestion methods for elemental determinations in plant material by ICP techniques, Analytica Chimica Acta378 191–200.

Shikov A. N., Pozharitskaya O. N., Makarov V. G., Wagner H., Verpoorte R., Heinrich M., 2014. Medicinal Plants of the Russian Pharmacopoeia; their history and applications Journal of Ethnopharmacology 154 481–536.

Stefkov G., Miova B., Dinevska-Kjovkarovska S., Petreska Stanoeva J., Stefova M., Petrusevska G., Kulevanova S. 2014 Chemical characterization of Centaurium erythrea L. and its effects on carbohydrate and lipid metabolism in experimental diabetes Journal of Ethnopharmacology 152(1), 71–77.

Suchacz B., Wesołowski M., 2006. The recognition of similarities in trace elements content in medicinal plants using MLP and RBF neural networks. Talanta 69 37–42

Subotić A, Jevremović S, Trifunović M, Petrić M, Milošević S, Grubišić D., 2009 The influence of gibberellic acid and paclobutrazol on induction of somatic embryogenesis in wild type and hairy root cultures of Centaurium erythraea—Gillib. African Journal of Biotechnology 8:3223–3228

Van Rossum, F., 2009. Succession stage variation in population size in an early- successional herb in a peri-urban forest. Acta Oecol. 35, 261–268.

Wada S., 2004 What are Trace Elements? - Their deficiency and excess states. JMAJ 47(8): 351–358, 2004

WHO, Guidelines for Assessment of Herbal Medicines, Geneva, 1991

Michaela Zeiner, Iva Juranovi´ c Cindri´ c, Martina Poˇ zgaj, Raimund Pirkl, TeaˇSili´ c,

Gerhard Stingeder Influence of soil composition on the major, minor and trace metal content of Velebit biomedical plants. Journal of Pharmaceutical and Biomedical Analysis 106 (2015) 153–158

Chunhong Zhanga, QianquanLia,b, MuxinZhangc, NaZhanga, MinhuiLia Effects of rare earth elements on growth and metabolism of medicinal plants Acta Pharmaceutica Sinica B 2013;3(1):20–24

Zinicovscaia, I. Ciocarlan, A. Lupascu, L. Aricu, A. Dragalin, I. Ciocarlan, N. Yushin. N. Investigation of elemental content and antimicrobial activity of Tanacetum corymbosum (L.) Sch. Bip. Journal of Radioanalytical and Nuclear Chemistry 2019, 321(1), 349-354

Zinicovscaia, I.Gundorina, S. Vergel, K. Grozdov, D. Ciocarlan, A. Aricu, A. Dragalin, I. Ciocarlan. N.Elemental analysis of Lamiaceae medicinal and aromatic plants growing in the Republic of Moldova using neutron activation analysis. Phytochemistry Letters, 35 (2020), 119-127, https://doi.org/10.1016/j.phytol.2019.10.009

Pavlov, S.S., Dmitriev, A.Yu., Frontasyeva, M.V., 2016. Automation system for neutron activation analysis at the reactor IBR-2, Frank Laboratory of Neutron Physics, Joint Institute for Nuclear Research, Dubna, Russia. Journal of Radioanalytical and Nuclear Chemistry, 309, 27-38.

Figure captions:

Figure 1 Map showing the study areas and sampling locations: ■ - lignite basin sites, □ – urban sites, ○ – rural sites.

Funding:

This study was carried out in the frame of the Joint Institute for Nuclear Research Programme (JINR-Poland).
