# Supplementary material for: Nuclear analytical techniques used to study the trace element content of Centaurium erythraea Rafn, a medicinal plant species from sites with different pollution loads in Lower Silesia (SW Poland)
Source: PLoS One. 2023 May 18;18(5):e0285306. doi: 10.1371/journal.pone.0285306 (PMC10194893; doi:10.1371/journal.pone.0285306)
Supplement: Attachment — Submitted filename: Response to Reviewers.doc [file pone.0285306.s002.doc]

*The Editor of Plos One	21* February 2022

Dear Editor,

Please find corrected enclosed an original manuscript entitled *“Nuclear analytical techniques used to study the trace element content of* Centaurium erythraea *Rafn, a medicinal plant from sites with different pollution loads in Lower Silesia (SW Poland)”* by Anna Brudzińska-Kosior, Grzegorz Kosior, Monika Sporek, Zbigniew Ziembik, Inga Zinicovscaia, Marina Frontasyeva, Agnieszka Dołhańczuk-Śródka.

Manuscript was corrected according to reviewers suggestions:

Reviewer: The submitted file does not contain tables. Since they were submitted as "Other", they have never reached reviewers. In the revised file, please embed tables into the main text or submit them as "Supporting material". **done**

Reviewer: The language usage can be improved. I strongly encourage the authors to go throughout the text once again, preferably along with a native English speaker, to correct syntax, interpunction, and typos. **The text has been checked for language by a native speaker**

Reviewer: Also note that the species authority is written Rafn (without full stop) as it refers to Carl Gottlob Rafn, a Danish botanist. **done**

Reviewer: References regarding biological activities of C. erythraea are quite outdated. Please check for updates.

**Unfortunately, the literature on the species studied is extremely scarce, therefore we used the current literature on medicinal plants.**

Your's sincerely

Grzegorz Kosior
Institute of Environmental Engineering and Biotechnology
Opole University
ul. Kard. Kominka 6a, 45-035 Opole
tel: +48/781797137
