# Supplementary material for: Nuclear analytical techniques used to study the trace element content of Centaurium erythraea Rafn, a medicinal plant species from sites with different pollution loads in Lower Silesia (SW Poland)
Source: PLoS One. 2023 May 18;18(5):e0285306. doi: 10.1371/journal.pone.0285306 (PMC10194893; doi:10.1371/journal.pone.0285306)
Supplement: Attachment — Submitted filename: Response to Reviewers.doc [file pone.0285306.s003.doc]

*The Editor of Plos One	21* October 2022

Dear Editor,

Please find corrected enclosed an original manuscript entitled *“Nuclear analytical techniques used to study the trace element content of* Centaurium erythraea *Rafn, a medicinal plant from sites with different pollution loads in Lower Silesia (SW Poland)”* by Anna Brudzińska-Kosior, Grzegorz Kosior, Monika Sporek, Zbigniew Ziembik, Inga Zinicovscaia, Marina Frontasyeva, Agnieszka Dołhańczuk-Śródka.

Manuscript was corrected according to reviewers suggestions:

Reviewer #3:The statistical analysis and the methodology are well done. However, the safety assessment part (line 271-276) might need to be improved due to the outdated references; WHO (1991) and Markert (1992); and the lack of criteria, as only the permissible level of As, Cd and Pb were indicated. Therefore, I recommend including additional permissible levels, such as from national regulations where the study was conducted or from regional regulations such as in Europe or the United States. This might help to consider the usage of this plant on an international scale.
Moreover, the consumption rate of medicinal plants is also an important factor. ***Thank you very much for submitting your comments.We have added appropriate corrections.***

I suggest calculating some trace elements toxicity assessment indices that involved consumption rate such as Daily Intake of Metal (DIM) and Health Risk Index (HRI). These indices could help the readers to assess the safety of most metals. The consumption rate could be adapted from previous studies or by assumption to recommend the optimal consumption rate. ***This is a medicinal plant, this plant material is not consumed every day, so we do not see the application of the above-mentioned factors.***

Reviewer #4: Dear Authors,
Its my pleasure to review your research and suggest comments aimed for its wider acceptability to researchers and scientists working in the field. I find the work novel and interesting. Few comments,
1. Abstract- Line number 30: 'reference plant' is a confusing term. What this implies? LIne number 30-31, need to be revised.
2. Abstract is vague. What are the investigated elements? Try to follow a model- 1-2 lines on background/need for the study, 2 lines on methodology, 3-4 lines on result and discussion (with some important data quotes from the study) and lastly 1 line on strong conclusion/recomendation. I will suggest the authors to reframe the abstract.
3. Introduction- My suggestion will be to bring the context from a 'global' to 'local' scale gradually, as readers may be from different geographical locations working on some other plant model but the conceptualisation of the research problem may be similar to the study presented. The most recent reference mentioned in the Introduction part is of Ziener et al., 2015; Hamza et al., 2015. This is not acceptable as per my view. Recent references need to be quoted. Globally there has been plethoras of work on pollution and its impact on ethnopharmacologically significant species- <https://link.springer.com/article/10.1007/s11356-021-12566-w>, <https://link.springer.com/article/10.1007/s10653-020-00603-5>, <https://link.springer.com/article/10.1007/s10661-019-7714-7>, <https://www.tandfonline.com/doi/abs/10.1080/15226514.2018.1524841> etc.

***Thank you very much for submitting your comments.We have added appropriate corrections.The text has been checked for language by a native speaker.***

Your's sincerely

Grzegorz Kosior
Institute of Environmental Engineering and Biotechnology
Opole University
ul. Kard. Kominka 6a, 45-035 Opole
tel: +48/781797137
