# Supplementary material for: Nuclear analytical techniques used to study the trace element content of Centaurium erythraea Rafn, a medicinal plant species from sites with different pollution loads in Lower Silesia (SW Poland)
Source: PLoS One. 2023 May 18;18(5):e0285306. doi: 10.1371/journal.pone.0285306 (PMC10194893; doi:10.1371/journal.pone.0285306)
Supplement: Attachment — Submitted filename: Response to Reviewers.doc [file pone.0285306.s004.doc]

*The Editor of Plos One	15* January 2023

Dear Editor,

Please find corrected enclosed an original manuscript entitled *“Nuclear analytical techniques used to study the trace element content of* Centaurium erythraea *Rafn, a medicinal plant from sites with different pollution loads in Lower Silesia (SW Poland)”* by Anna Brudzińska-Kosior, Grzegorz Kosior, Monika Sporek, Zbigniew Ziembik, Inga Zinicovscaia, Marina Frontasyeva, Agnieszka Dołhańczuk-Śródka.

Manuscript was corrected according to reviewers suggestions:

***Thank you very much for submitting your comments. We agree with all of the reviewers' suggestions and have made major changes to the text to meet your requests.***

Your's sincerely

Grzegorz Kosior
Institute of Environmental Engineering and Biotechnology
Opole University
ul. Kard. Kominka 6a, 45-035 Opole
tel: +48/781797137
